# Advanced Imaging in Femoroacetabular Impingement: Current State and Future Prospects

**DOI:** 10.3389/fsurg.2015.00034

**Published:** 2015-07-24

**Authors:** Bernd Bittersohl, Harish S. Hosalkar, Tobias Hesper, Carl Johan Tiderius, Christoph Zilkens, Rüdiger Krauspe

**Affiliations:** ^1^Department of Orthopedics, Medical Faculty, University Düsseldorf, Düsseldorf, Germany; ^2^Center for Hip Preservation and Children’s Orthopedics, San Diego, CA, USA; ^3^Department of Orthopedic Surgery, Lund University Hospital, Lund, Sweden

**Keywords:** hip, femoroacetabular impingement, cartilage, MRI, dGEMRIC, T1rho mapping, T2 mapping, T2* mapping

## Abstract

Symptomatic femoroacetabular impingement (FAI) is now a known precursor of early osteoarthritis (OA) of the hip. In terms of clinical intervention, the decision between joint preservation and joint replacement hinges on the severity of articular cartilage degeneration. The exact threshold during the course of disease progression when the cartilage damage is irreparable remains elusive. The intention behind radiographic imaging is to accurately identify the morphology of osseous structural abnormalities and to accurately characterize the chondrolabral damage as much as possible. However, both plain radiographs and computed tomography (CT) are insensitive for articular cartilage anatomy and pathology. Advanced magnetic resonance imaging (MRI) techniques include magnetic resonance arthrography and biochemically sensitive techniques of delayed gadolinium-enhanced MRI of cartilage (dGEMRIC), T1rho (T1ρ), T2/T2* mapping, and several others. The diagnostic performance of these techniques to evaluate cartilage degeneration could improve the ability to predict an individual patient-specific outcome with non-surgical and surgical care. This review discusses the facts and current applications of biochemical MRI for hip joint cartilage assessment covering the roles of dGEMRIC, T2/T2*, and T1ρ mapping. The basics of each technique and their specific role in FAI assessment are outlined. Current limitations and potential pitfalls as well as future directions of biochemical imaging are also outlined.

## Introduction

Seemingly, first described by Smith-Peterson in 1936 (1) and then in more detail by Stulberg et al. (2), Harris (3), and Ganz et al. (4), femoroacetabular impingement (FAI) refers to a condition in which structural abnormalities of the proximal femur and/or acetabulum lead to mechanical abutment or conflict during hip motion. Pain, loss of function, and restriction of motion are characteristic symptoms. Moreover, symptomatic FAI has now been recognized as a cause of early osteoarthritis (OA) of the hip (5, 6). The exact pathomechanism and the threshold including the time frame and severity of this abutment that eventually results in irreversible degeneration of the hip joint remain an enigma.

**Figure 1:**
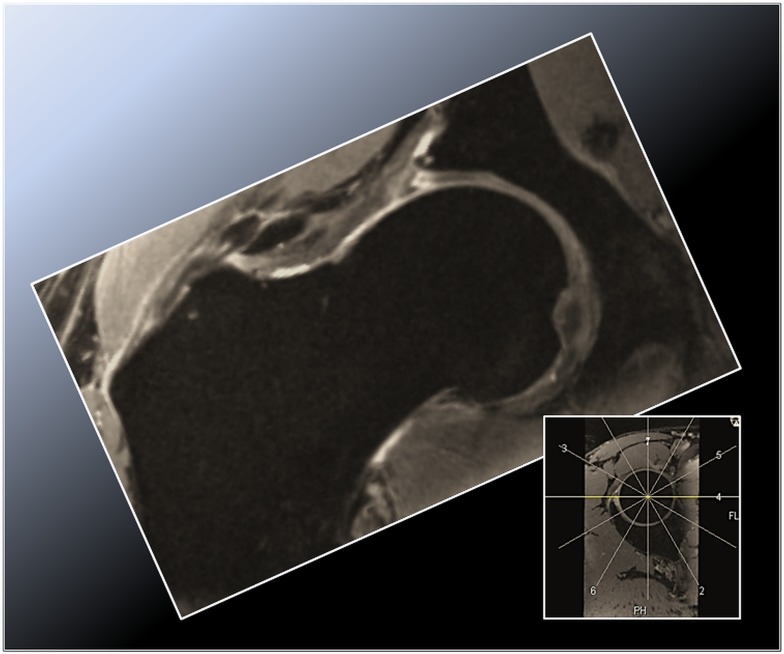
**Radial double-echo steady state (DESS) reformat depicting the superior zone (12 o’clock position) in a cam-type FAI hip**. Note the aspherical femoral head and the corresponding labral tear with intraosseous and extraosseous extravasation of synovial fluid arising from the torn labrum and peripheral acetabular cartilage abrasion.

Femoroacetabular impingement is classified as *cam*-type when the abutment is triggered by an aspherical femoral head that generates shearing forces against the anterosuperior acetabular rim structures while entering the joint during hip flexion and internal rotation (4, 5). Labral tears, cartilage abrasion, and cartilage delamination from the labrum and subchondral bone can result from cam impingement (Figure 1). Cartilage delamination may occur without the disruption extending through the cartilage surface (referred to as the carpet phenomenon because of its similarity to a carpet on a greasy floor). Disruption extending to the cartilage surface creates a flap tear. Cam-type FAI is common in young men. An osseous asphericity (“bump”) located along the anterosuperior aspect of the femoral head–neck junction may appear as “pistol grip” in an anteroposterior (AP) radiograph.

**Figure 2:**
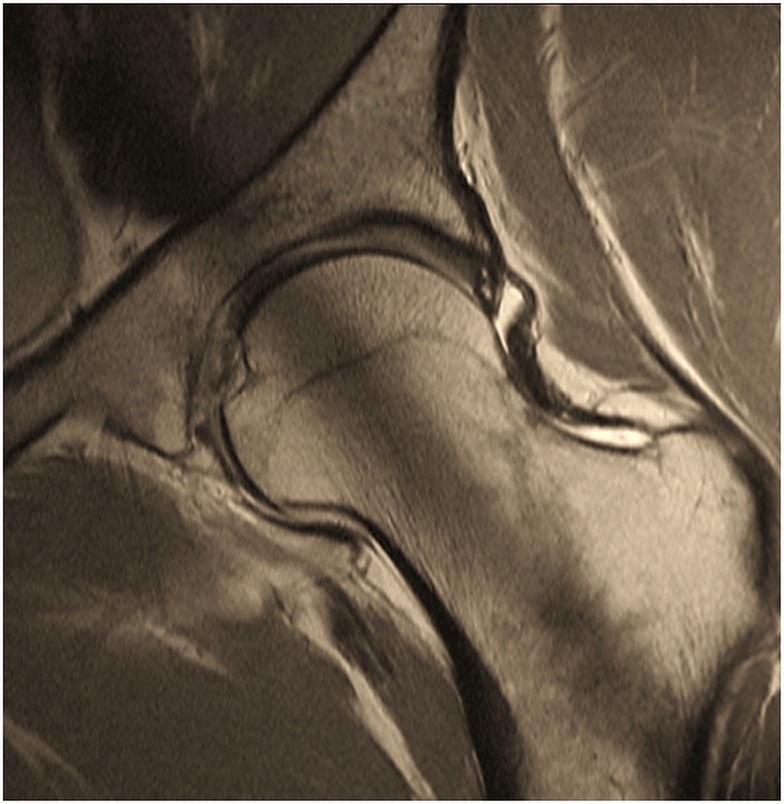
**Two-dimensional proton-density (PD) – weighted MR image of a pincer-type FAI patient depicting an increased signal within the center of the labrum that does not extend to the labral margin reflecting intra-labral degeneration**. Note that the saturation effect (band of low signal in the center of acetabulum and femoral neck) is constantly present in 2D radial MR imaging.

In *pincer*-type FAI, the abutment of the femoral neck against the acetabulum results from over coverage by the acetabulum (4, 5). The extent of femoral head coverage with abutment may be focal (loss of normal cranial acetabular anteversion, i.e., focal relative retroversion; identified radiographically as a “cross-over sign”) or global (increased lateral or anterior center-edge angles, posterior wall sign, prominent ischial spine sign). A deep acetabulum (coxa profunda) with or without femoral head medialization (protrusio acetabulae) may variable culminate in pincer-type FAI. Notably, the cross-over sign has recently been challenged as an accurate measure of cranial acetabular version, as the anterior inferior iliac spine is superimposed and may account (falsely elevate) for a large proportion of positive cross-over signs. A hypertrophied and deformed labrum, labrum ossification, and labral tearing with (succeeding) linear cartilage damage are somewhat distinctive observations in pincer-type impingement (Figure 2). A chondral contrecoup lesion at the posteroinferior aspect of the hip joint owed to a lever mechanism at the anterior acetabular rim (during flexion the femoral head can be levered against the posterior wall of the acetabulum, causing shear forces on the posterior chondral surfaces) is another common finding. The pattern of chondrolabral damage in pincer FAI, which is common in middle-aged women, may be circumferential. However, most lesions occur at the anterosuperior acetabular rim as flexion is the central movement of the hip. Notably, many patients reveal morphological FAI features on both sides of the hip joint (then referred to as mixed-type impingement). Whether these features are the normal continuum of initial isolated cam or pincer lesions or a unique bilateral morphology in themselves remains largely unknown.

Femoroacetabular impingement remains a clinical diagnosis that is re-affirmed with imaging. Although cam- and pincer-FAI morphologic features are currently interpreted somewhat variably on imaging modalities (for example, varying threshold values for measuring the aspherity of the femoral head), it is important to note that incidental radiographic findings suggestive of FAI morphology are commonly reported even when individuals are asymptomatic (reported prevalence of an asymptomatic cam deformity of 37 and 67% of an asymptomatic hip with pincer deformity) (7). Having identified the classical physical examination findings, radiographic imaging aims (1) to identify the morphology leading to abutment in the individual case and thus confirm the radiographic diagnosis of FAI, (2) to define the pathological extent of the impingement, (3) to evaluate the extent and severity of chondrolabral damage at the time of presentation, and (4) to differentiate other relevant diagnoses that may occasionally co-exist, including labral tears with hip dysplasia. A variety of AP and lateral plain radiographs and magnetic resonance imaging (MRI) or MR arthrography (MRA) are the primary imaging modalities (8–10). The radiographs provide initial information about the osseous structural abnormalities of the hip and allow a comparison of the affected side with the asymptomatic side for the detection of subtle osseous changes pointing toward morphology of FAI. With superior soft tissue contrast and the capacity for multi-planar image acquisition, MRI and MRA can reveal the degree of chondrolabral damage. In addition, they provide crucial information on the location and extent of hip deformity and other causes of hip pain (such as avascular necrosis of the femoral head, neoplastic synovitis) can be excluded. If surgical treatment is intended, pre-operative MRI or MRA assists in identifying the degree of cartilage damage that may otherwise negatively affect the surgical outcome (11). The utility of contrast agents (MRA) or diagnostic anesthetic into the hip joint (to confirm intra-articular pathology by artificially creating an arthrogram effect) simplifies evaluation by separating the intra-articular structures to delineate the anatomy better (12). Furthermore, the high signal of gadolinium and joint fluid can be visualized clearly in any surface irregularity if present. Computed tomography (CT) and CT arthrography may be used (in patients with contraindications to MRI) because they can offer a three-dimensional (3D) display of the osseous anatomy and sequelae of impingement (13). The 3D assessment helps to define the nature, location, and extent of femoral head over-coverage or femoral head–neck prominence. With a diagnosis on clinical examination, the correct implementation of the various imaging techniques is critical in the evaluation of morphology, deformity evaluation, and planning of management.

**Figure 3:**
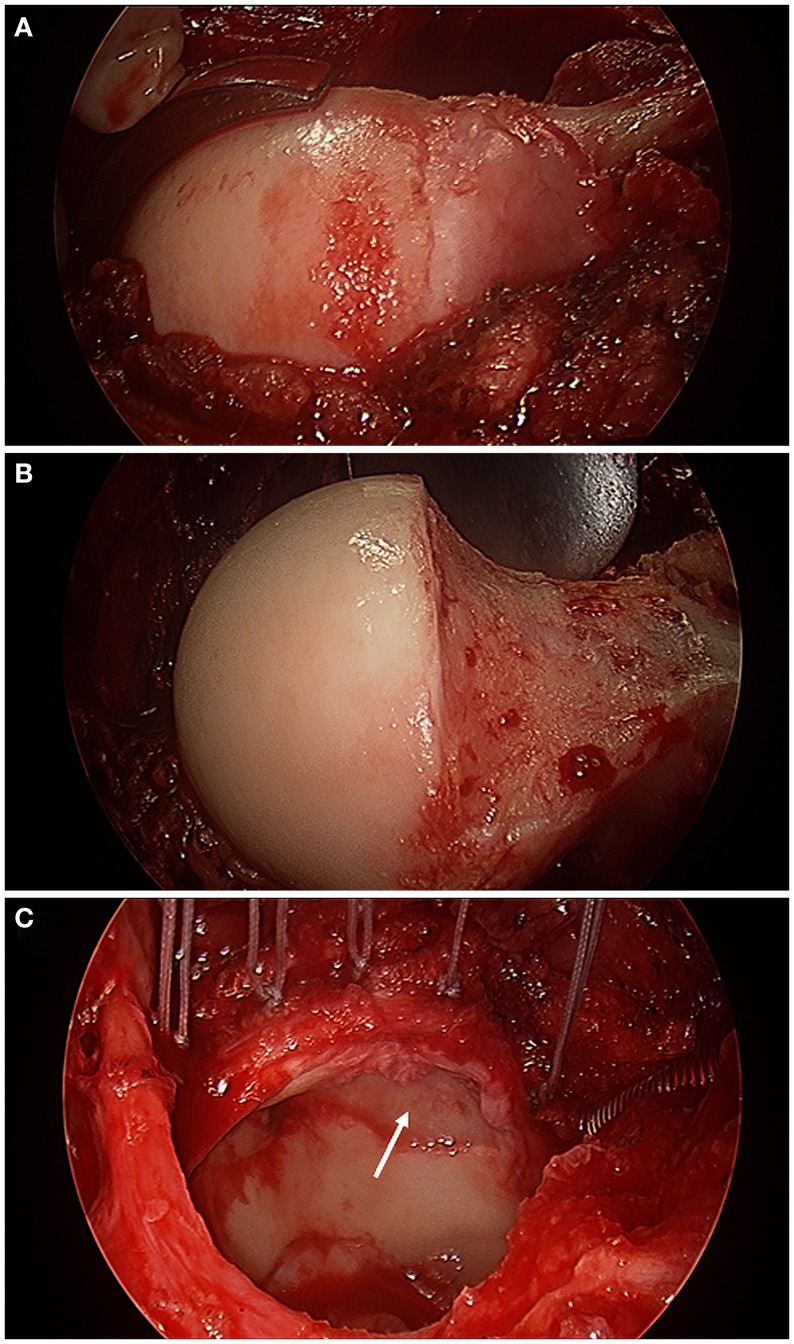
**Intra-operative photographs made with an arthroscopic surgery camera after surgical hip dislocation demonstrating a bump deformity at the femoral head–neck junction (A), the femoral head–neck osteochondroplasty to improve the femoral head–neck offset (B), and acetabular evaluation under full direct visualization revealing full-thickness chondral damage at the anterior–superior aspect of the acetabulum (white arrow) and an extended torn labrum that was re-attached to the acetabular rim with five suture anchors (C) in a 56-year-old with impingement**.

The therapeutic goal in symptomatic FAI is to address the abnormal morphology, that is, responsible for the impingement in that individual case, thereby to mitigate the course of progression to arthritis. Pain relief and improvement of motion and function are often realized following the achievement of de-impingement. Recent advances also aim to address and treat chondrolabral lesions in many different ways in order to stop or at least slow the progress of degenerative OA. Depending on the pattern of FAI, the extent of pre-existing chondrolabral damage, the patient’s expectations, and the surgeon’s training, a number of surgical treatment options are possible (14). These range from hip arthroscopy to mini-open arthrotomy, a combined open arthrotomy – arthroscopic procedure and surgical hip dislocation with appropriate management of intra-articular damage. Depending on the intra-operative observation, debriding or repair of any pre-existing chondrolabral pathology and concomitant femoral head–neck or acetabular osteochondroplasty to improve the femoral head–neck offset is indicated (Figure 3). In selected cases, acetabular or femoral correction osteotomies may also be necessary. Recent advances include chondrocyte grafting and chondrocyte transplantation in select cases (15).

A successful outcome following surgical treatment certainly includes the basic requirement of correcting the deformity of abnormal morphology in that individual case. There is no question that the preceding chondrolabral cartilage damage is a strong predictor of the eventual outcome of surgery, often producing poor outcomes in cases with cartilage degeneration in the advanced stages (16). Identification of patients with FAI in the early phases of chondrolabral damage and timely surgical intervention prior to the onset of progressive irreversible chondral damage is critical to the long-term success of FAI treatment. Conversely, despite technical developments that include the use of high-MR field strengths and dedicated cartilage-specific sequences, a comprehensive pre-operative assessment of hip-joint cartilage is still challenging given its location deep within the body, its thinness and its spherical shape, which requires both high-spatial resolution and a high-signal-to-noise (SNR) ratio (17). Also, in FAI cartilage, damage occurs typically as a debonding of the acetabular cartilage from the subchondral bone, leaving the superficial layer intact (5). Therefore, as the contrast medium in MRA will usually not penetrate beneath delaminated cartilage, the extent of the acetabular cartilage damage is probably underestimated in many cases (18). Hence, the accuracy and reliability achieved with MRI and MRA in identifying early chondral damage in FAI remain rather poor (19, 20). However, the accuracy and diagnosis achieved by MRI/MRA are technique dependent (21). Notably, the sensitivity of detection of cartilage delamination, for example, the revealing of fluid under cartilage tissue, has been proved to be at best moderate (sensitivity rates in one recent study range from 35 to 74%) (22).

Biochemically sensitive MRI techniques may help to overcome this limitation as they reproducibly quantify extracellular matrix alterations within cartilage that occur early in the progress of cartilage degeneration prior to advanced changes or gross morphological damage. Biochemically sensitive MRI includes the techniques of delayed gadolinium-enhanced MRI of cartilage (dGEMRIC), T1ρ (T1rho), T2/T2* mapping, and several others (23). The ability of these techniques to evaluate cartilage degeneration accurately and reproducibly could improve the ability to offer fairly reliable and predictable prognostication of whether a patient would benefit from joint preservation surgery for symptomatic FAI.

The present review aims to outline the facts and current applications of biochemical MRI for hip joint cartilage assessment covering the roles of dGEMRIC, T2/T2*, and T1ρ mapping. Therefore, the basics of each technique and potential implications for patient care in FAI are outlined. Furthermore, current limitations and potential pitfalls and the present and future aspects of biochemical MRI in FAI are discussed.

## Delayed Gadolinium-Enhanced MRI of Cartilage

Delayed gadolinium-enhanced MRI of cartilage is sensitive to the negative charge of the extracellular glycosaminoglycan (GAG) in which the negatively charged gadolinium-based contrast agent distributes within cartilage inversely to the GAG content (24). Thus, regions with diseased cartilage will demonstrate larger amounts of gadolinium and vice versa. Contrast agent reduces the T1 relaxation time. Thus, higher T1_Gd_ relaxation time values will be measured in healthier cartilage, whereas low T1_Gd_ values will be observed in degenerated, GAG-depleted cartilage.

Most dGEMRIC studies have been performed with the FDA-approved, intravenously injected double negatively charged contrast agent Gd-DTPA^2−^. Although, more recently, the single negatively charged contrast agent Gd-DOTA^−^ has been used both after intravenous (25) and after intra-articular administration (26), providing the benefits of both MRA and cartilage mapping. The suggested contrast media dosage for a dGEMRIC measurement is 0.2 mm/kg body weight, twice the recommended clinical dose (27). A definite time frame between the contrast agent administration prior to an exercise protocol and the T1_Gd_ relaxation time measurement, which is based on the route of administration (intravenous or intra-articular) and the thickness of the cartilage tissue (longer uptake times in knee joint cartilage), is required to ensure appropriate penetration of the gadolinium contrast agent into cartilage. For dGEMRIC of hip joint cartilage, a time frame between contrast agent administration and T1_Gd_ relaxation time measurement of 30–90 min after the intravenous application (27) and 15–30 min after the intra-articular injection (28) has been proposed. Notably, diseased cartilage may reveal a faster gadolinium wash-in into cartilage, indicating that T1_Gd_ mapping at earlier time points (after 30–65 min, for instance) may increase sensitivity to cartilage alterations (29).

**Figure 4:**
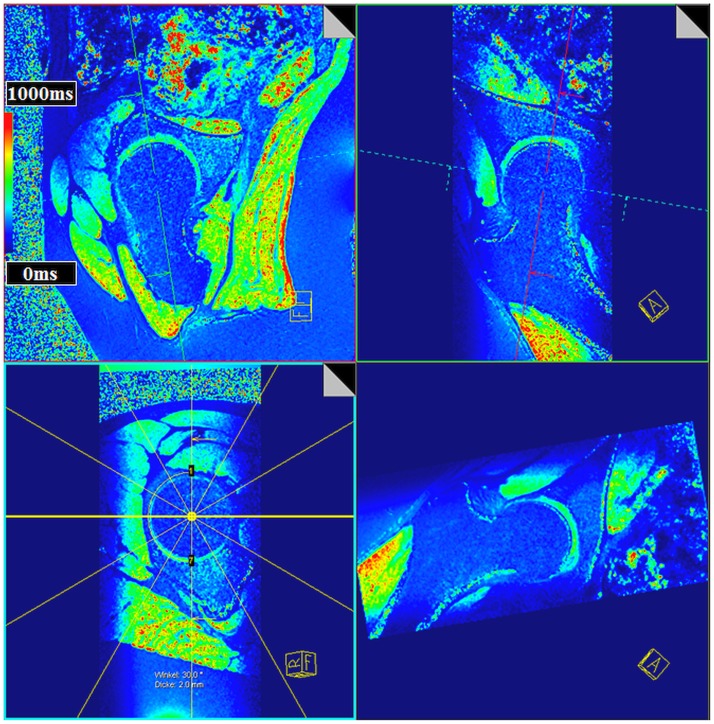
**Multi-planar-reconstruction of the three-dimensional (3D) T1_Gd_ data set including plane adjustment through the center of the femoral head and perpendicular to the femoral neck within the sagittal oblique view and the coronal oblique view to create radial T1_Gd_ planes throughout the hip joint**. T1_Gd_ values are visualized in a color scale. Note the aspherical nature of the femoral head of this asymptomatic volunteer yet without a decrease in the T1_Gd_ values indicating a normal GAG content within cartilage.

For generating a T1 relaxation time image (T1_Gd_ after gadolinium contrast application), consecutive images with varying repetition times (TR) and signal levels are required. T1_Gd_ maps were initially obtained with two-dimensional (2D) T1-weighted inversion recovery (IR) sequences that offered the advantages of widespread availability, optimal contrast properties, and relatively low B1 variation, which arise because the radiofrequency (RF) pulse is absorbed differently across the patient, particularly in a high-MRI field (30). Explanatory note: in MRI, there are three types of magnetic fields including the main magnetic field (B0), the RF field that excites the spins (B1), and the gradient fields that offer localization. The main limitations of this 2D-based technique include longer acquisition time and risk of motion artifacts (31). Current techniques, such as gradient-echo (GRE), -based sequences with variable flip angles are capable of generating 3D T1_Gd_ data sets with high-isotropic spatial resolution. These 3D MRI data sets can then be reformatted during post-processing in radial planes of the hip joint (Figure 4) instead of just a selected cross-section as with 2D T1_Gd_ mapping (32). Although 3D dGEMRIC is relatively new, recent investigations confirm that it is both highly reproducible and valid in its assessment of hip articular cartilage (33–36). Lattanzi et al. have established a new high resolution, B1-insensitive 2D T1 mapping saturation and recovery pulse sequence with fast spin-echo readout for dGEMRIC of the hip at 3 T including radial imaging (37).

### Literature review

Jessel et al. noted a correlation between the T1_Gd_ value and pain (regression coefficient of 0.4; *P* < 0.05) and between the T1_Gd_ value and the alpha angle (coefficient of 0.36; *P* < 0.05), which is a parameter for calculating the asphericity at the femoral head–neck junction (38). Although the amount of radiographic apparent OA was mild (Tönnis grade 0 or 1) in the majority of cases (26 of 37 hips), the drop in T1_Gd_ (T1_Gd_: 464 ± 64 ms) was remarkable. Notably, neither Tönnis grade nor joint space width correlated with patient symptoms.

**Figure 5:**
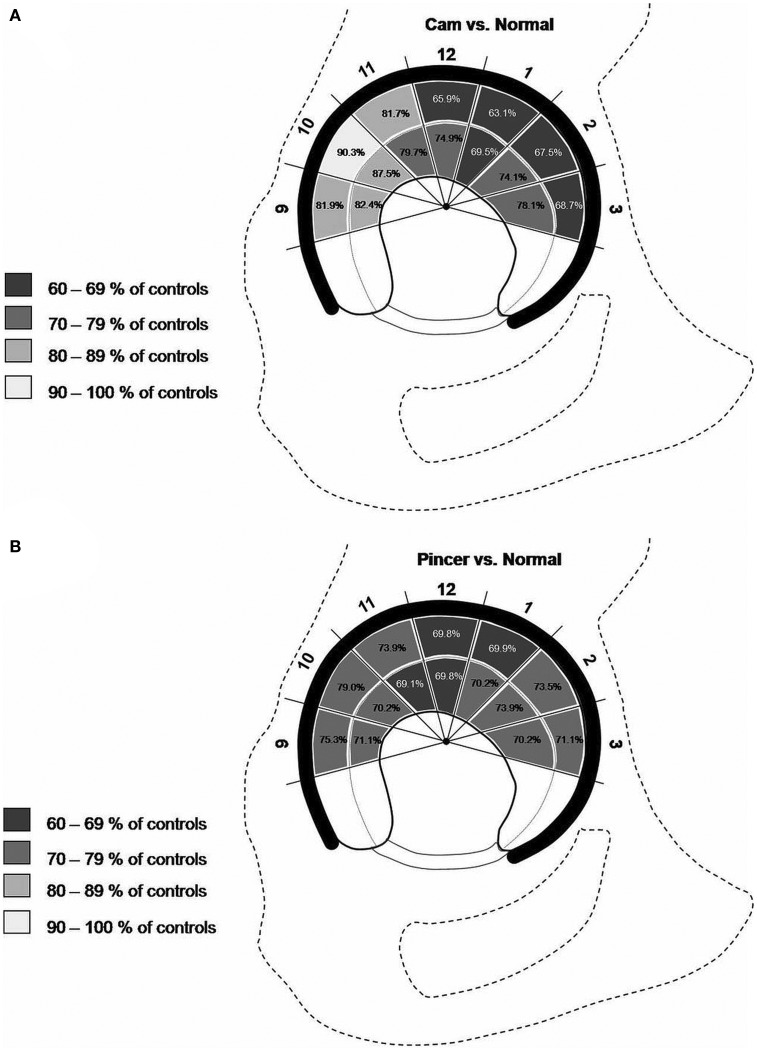
**Schematic drawing demonstrating the T1_Gd_ decrease in various regions of hip joint cartilage of cam- (A) and pincer- (B) FAI patients**. The percentage values refer to the T1_Gd_ average in corresponding hip joint regions of asymptomatic controls. Note that, particularly in the anterior aspect of the joint, the cam-FAI group exhibited not only a peripheral but also a central cartilage T1_Gd_ decrease **(A)**, whereas the pincer-FAI cohort demonstrated a rather global T1_Gd_ decrease for all areas of the hip **(B)**. Figure reprinted with permission (40).

Bittersohl et al. observed lower T1_Gd_ values in FAI patients in comparison with asymptomatic volunteers (39). Furthermore, the distribution of the T1_Gd_ decrease was in accordance with the FAI damage pattern, which in cam types demonstrated a significant drop of the T1_Gd_ values in the anterior to superior location (*P* < 0.05). In pincer-type FAI, a generalized circumferential decrease was noted. Mamisch et al. reported lower T1_Gd_ values in cam- and pincer-FAI patients than in asymptomatic controls (40). Particularly in the anterior aspect of the joint, the cam-FAI group exposed not only peripheral but also central cartilage T1_Gd_ changes, whereas the pincer-FAI cohort demonstrated a rather global T1_Gd_ decrease for all areas of the hip, with T1_Gd_ values between 69.1 and 79% of the control group (Figure 5). The results of these studies are somewhat similar to those of Domayer et al., who studied the T1_Gd_ pattern in symptomatic cases of hip dysplasia and FAI (41). Twenty patients with hip dysplasia and 20 patients with FAI underwent dGEMRIC. The mean T1_Gd_ value was 551 ± 95.7 ms in patients with FAI and 531 ± 92.7 ms in patients with hip dysplasia. In pre-arthritic hip joints (in this study defined by T1_Gd_ values >500 ms), higher T1_Gd_ values were noted in the weight bearing and in the central areas in both study cohorts (*P* = 0.036 and 0.0001), whereas no such distribution was noted in hips with progressive degeneration (T1_Gd_ values <500 ms). Notably, in view of the high content of GAG in the weight-bearing superior region, the regional distribution of T1_Gd_ in the hip joint with increased values toward the superior and central regions has been noted in asymptomatic adult volunteers (42). These observations regarding the T1_Gd_ pattern both in asymptomatic volunteers and in FAI patients (cam, pincer, and mixed types) may aid in objective stratification and treatment planning.

Pollard et al. spotted lower T1_Gd_ values in asymptomatic hips with cam deformities compared with morphologically normal hips (*P* = 0.0008) (43). The T1_Gd_ values in the anterosuperior aspect of the acetabular cartilage correlated inversely with the alpha angle (*r* = −0.483; *P* = 0.0038), indicating that the severity of the GAG loss correlates with the magnitude of the cam deformity. Furthermore, cases with a positive impingement test demonstrated lower global (total femoral and acetabular cartilage) T1_Gd_ values than hips with a negative result (T1_Gdtotal_ = 625 versus 710 ms; *P* = 0.0152). Somewhat similar observations were made by Jessel et al., who noted a weak correlation (*r* = −0.36) between the alpha angle and femoroacetabular T1_Gd_ value (38). Zilkens et al. noted a correlation between the beta angle (angle between the femoral head–neck junction and acetabular rim) in the superoinferior and superior regions, whereas the alpha angles did not correlate with the T1_Gd_ measures (44). Zilkens et al. explain their results by the fact that the alpha angle only reflects the femoral side, whereas the beta angle accounts for the morphology of both the femur and the acetabulum and thus may be the more sensitive surrogate for cartilage damage in FAI.

Despite several technical developments in recent years that have made dGEMRIC a clinically feasible application in the assessment of hip joint cartilage status, one should exercise care during interpretation of dGEMRIC observations prior to implementing any clinical decisions because anatomic, inter-subject, and technically related variations can lead to meaningful misinterpretations and limited comparability. The above-mentioned regional differences in GAG concentration, the effect of the magnetic field strength on the T1 relaxation time and pharmacokinetic-related contrast agent uptake variations owed to patient age, sex, body mass index (BMI), or differences in diffusion and transport rates of gadolinium contrast are just a few examples in this context. Lattanzi et al. therefore proposed a standardized approach to analyze dGEMRIC measurements in FAI (36). This included the transformation of T1_Gd_ values to standard scores (*z*) calculated from the mean and the SD of T1_Gd_ in the (in FAI) assumed healthy weight-bearing femoral head cartilage. Others proposed to normalize regional T1_Gd_ values by dividing them by the average T1 of the total cartilage (acetabular and femoral) to highlight areas of abnormalities (43).

## T1ρ Mapping

Similarly to dGEMRIC, T1rho (T1ρ) relaxation time mapping is sensitive to the GAG content of hyaline cartilage (45–49). The main advantage of T1ρ mapping is that it does not require an intravenous injection or an exercise regime or a time frame between contrast agent application and MRI to warrant gadolinium uptake into cartilage. However, a noticeable drawback of this technique is that it involves relatively high- RF energy [measured by the specific absorption rate (SAR)] and this high-RF energy can result in tissue heating during the spin-lock preparation pulse (50). Furthermore, the T1ρ sequence is, yet, not commercially available and still requires post-processing.

In brief (51–53), based on the physics of MRI, a 90° RF pulse is applied on-resonance with Larmor precession frequency to excite nuclei, meaning that spins are tilted in the main magnetic field B_0_ into the transverse plane and synchronized to spin (precess) in-phase. The synchronized precession of the spins in the transverse plane is the origin of an RF pulse (signal) that is collected in the MR receiver coil. Nuclei relaxation occurs immediately after the RF pulse because of the exchange of energy between the nuclei and their surroundings (spin–lattice or T1 relaxation) and from nuclei dephasing caused by variations in the precessing frequencies of the nuclei that arise from random interactions between adjacent nuclei (spin–spin or T2 relaxation). In GRE-MRI, which lacks a 180° spin-refocusing pulse, a combination of T2 and “noise” caused by local field inhomogeneities related to differences in the magnetic susceptibility among various tissues, chemical shifts, gradients applied to perform spatial encoding, and main magnetic field heterogeneity is measured. This is referred to as T2* relaxation. A T1ρ pulse sequence applies a long-duration, low-power RF pulse to the transverse component of the magnetization vector. The applied B_1_ field attenuates the effect of dipole–dipole coupling, chemical exchange, and background gradients on the magnetization, meaning that the regular signal decay (T2* relaxation) is slowed to a time constant T1ρ that is referred to as spin–lattice relaxation in the rotating frame. In other words, the magnetization is, for the duration of the RF pulse, “spin-locked.” Having deteriorated the T2/T2* effects by means of the “spin-locking” pulse, the T1ρ decay results principally from interactions between protons and their surroundings with regard to articular cartilage reflecting interactions between water molecules and extracellular components, such as GAG chains, that restrict the motion of water molecules, which explains the increased T1ρ values in cartilage regions with depleted GAG.

There are some conflicting reports in terms of GAG content and its correlation with T1ρ relaxation (54). Notably, Keenan et al. reported that T1ρ relaxation time is inversely correlated with the GAG content in cartilage regions with normal T2 relaxation time (55), whereas other researchers (56, 57) observed focal areas of high- and low-T1ρ and T2 values, which cannot be explained by GAG concentration or collagen orientation. Further conflicting evidence regarding the contribution of factors behind the variations in T1ρ and T2 is reported in the literature. However, it has been agreed that these measures are sensitive to alterations in the extracellular composition and macromolecular structure and integrity (54). Although the T1ρ technique has been explored extensively in the knee (58–63) the application of T1ρ mapping to the hip joint (54, 64, 65) has been relatively limited, which is in part related to signal-to-noise (SNR) ratio constraints associated with the thin cartilage layers and the deeper location of this joint.

### Literature review

Early investigations of T1ρ relaxation time mapping in subjects with FAI demonstrated degenerative changes in acetabular and femoral cartilage before gross tissue loss was apparent (65). It was also noted that FAI patients display a different T1ρ distribution pattern across the thickness of the cartilage whereby the control group demonstrated a T1ρ value trend with increasing values from deep to superficial cartilage layers, with the middle third having significantly greater T1ρ relaxation values than the deepest third (*P* = 0.008), whereas the FAI group demonstrated loss of this trend. Furthermore, the deepest third cartilage layers in the FAI group demonstrated greater T1ρ relaxation values than controls (*P* = 0.028).

**Figure 6:**
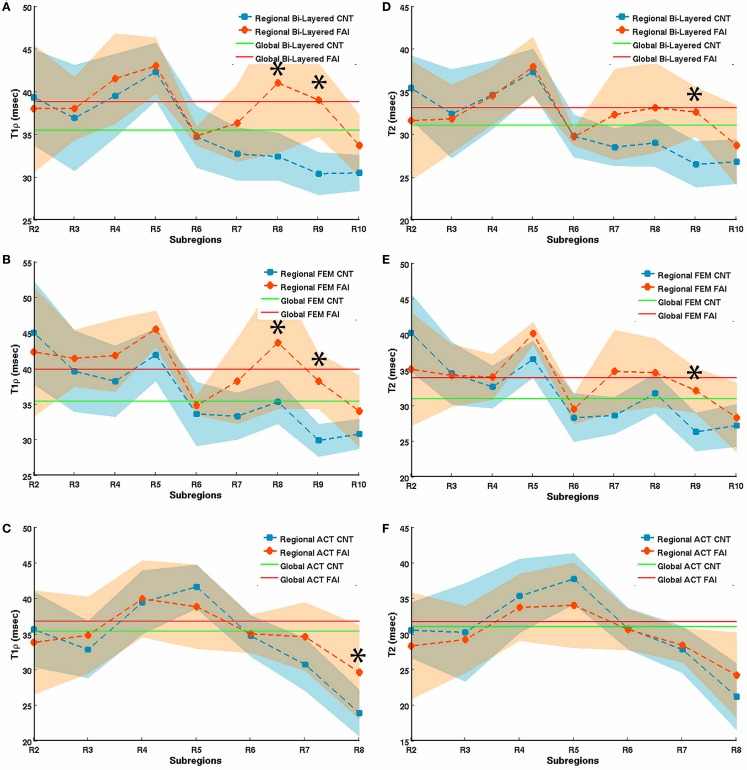
**T1rho (A–C) and T2 (D–F) relaxation times in bi-layered (A,D), femoral (B,E), and acetabular (C,F) sub-regions of hip joint cartilage in 12 healthy volunteers (controls) and 9 FAI patients**. The segmented regions of interest (ROIs) were automatically divided radially into 12 equal sub-regions (30° intervals) based on the fitted center of the femur head in which R2 represents the posterior–inferior region (5–4 o’clock in a clockwise system, right hip), R5 the posterior–superior (2–3 o’clock), and R8 the anterior–superior (1–2 o’clock) region. Error bars represent SD. *represents a significant difference between relaxation times of controls and FAI subjects. Figure reprinted with permission (54).

Using a 3-T MR scanner, Subburaj et al. noted longer T1ρ relaxation times (T1ρ = 39.9 ± 3.3 versus 35.4 ± 2.3 ms; *P* = 0.0020) and longer T2 relaxation times (T2 = 33.9 ± 3.1 versus 31.1 ± 1.7 ms; *P* = 0.0160) in the cartilage of 9 FAI patients than in 12 healthy controls (54). The authors also noted that T1ρ and T2 relaxation times in the anterosuperior cartilage sub-region were different from those of the global cartilage, and that the analysis based on local regions was more sensitive than global measures in differentiating subjects with and without FAI (Figure 6). Notably, the *in vivo* hip cartilage T1ρ and T2 measurements were highly reproducible (CV < 5%).

## T2 Mapping

Probing the interactions between water molecules and their environment, T2 relaxation time mapping is sensitive to two main components of articular cartilage, collagen, and water (66). It has been shown to correlate with cartilage matrix hydration and collagen fiber integrity whereby early degeneration-induced alterations in water content and collagen fiber arrangement could then be detected by this technique (T2 relaxation time increase) (67, 68). There has been a considerable amount of work on non-contrast-based assessment of early cartilage degeneration using T2 mapping. However, most of these studies relate to the assessment of knee joint cartilage (69) and only a few studies report the application of T2 mapping for the evaluation of hip joint cartilage. This is probably related to long-acquisition times that typically exceed 10 min, and the constraint on 2D acquisitions.

### Literature review

**Figure 7:**
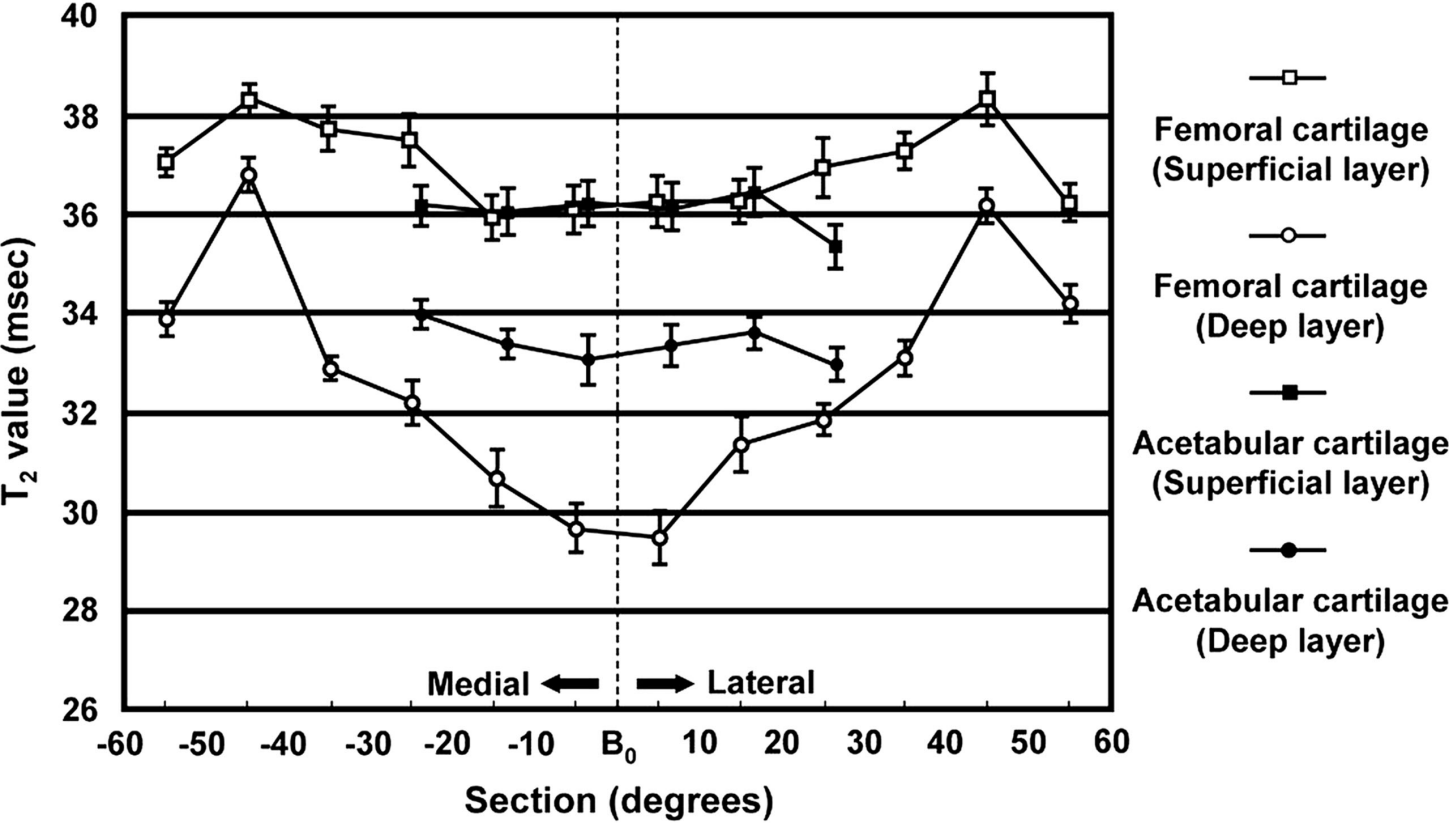
**T2 mean values in various of sections (regions) and layers (zones) of femoral and acetabular cartilage**. The bar indicates the SE of the mean. Note the topographic variation in the T2 values of hip joint cartilage probably because of factors including cartilage matrix composition and magic angle effect that need to be considered when interpreting and evaluating T2 values in hip joint cartilage. Figure reprinted with permission (70).

Probably because of factors including cartilage matrix composition and magic angle effect, Watanabe et al. (70) noted a topographic variation in the T2 values of hip joint cartilage of 12 healthy volunteers (Figure 7). These observations are of great relevance for interpreting and evaluating T2 values in hip joint cartilage before attributing T2 changes to early degeneration. Furthermore, the effect of cartilage compression during loading, which induces water outflow and derangement of the collagen organization, and hence, a decrease of T2 needs to be considered. For that reason, it is recommended to perform T2 mapping at the end of an MR scan to minimize the effects of cartilage loading. Interestingly, Nishii et al., who evaluated the change in cartilage T2 values with loading in 15 patients with hip dysplasia, noted that (1) the decrease in cartilage T2 at the outer superficial zones of the acetabular cartilage with loading was greater in patients with hip dysplasia (T2 change with loading: −7.6 ± 10.6%) than in healthy volunteers (T2 change with loading: −1.2 ± 10.9%) and (2) there was a positive correlation between the center-edge angle on AP radiographs and T2 changes with loading at the outer deep zones of the acetabular cartilage (71).

Ascani et al. studied the correlation of dGEMRIC and T2 with morphologic cartilage assessment at 3 T (72). Whereas the dGEMRIC technique was remarkably sensitive to cartilage damage (71 and 86% for minor and severe lesions, respectively), T2 mapping was very specific (87% for any type of lesion). The authors concluded that a combination of morphologic MRI, dGEMRIC, and T2 could be effective in detecting and staging cartilage damage. As outlined above, Subburaja et al. noted longer T2 relaxation times (T2 = 33.9 ± 3.1 versus 31.1 ± 1.7 ms; *P* = 0.0160) in cartilage of 9 FAI patients than in 12 healthy volunteers (54). Studies on other pre-arthritic hip conditions revealed similar results. Yamamoto et al. noted higher T2 values (T2 = 34.4 ± 3.1 versus 30.8 ± 1.2 ms; *P* = 0.001) of the femoral head cartilage in 10 systemic lupus erythematosus patients (15 hips) with non-collapsed osteonecrosis of the femoral head associated with corticosteroid therapy than in the control group (14 volunteers, 28 hips) (73). Nishii et al. observed a trend of higher T2 values (T2 = 37.1 ± 12.0 versus 33.4 ± 4.5 ms) in acetabular cartilage of 12 dysplastic hips with early (Kellgren–Lawrence grade 1 or 2) OA compared with a control group of 10 volunteers (14 hips) (74). Notably, whereas almost all hips of the control group (visually) demonstrated a characteristic gradient pattern of T2 with T2 values increasing from the deep cartilage zone toward the articular surface, which is consistent with previous reports of normal cartilage T2 values (75, 76), this cartilage T2 pattern became less apparent (pre-arthritic patients) or disappeared (early-arthritic patients).

## T2* Mapping

The T2* mapping technique is a recent modality that is relatively easy to implement in clinical routine as no contrast media or special hardware are required and it has the added advantage of short-acquisition times. Furthermore, high-resolution imaging allowing for a 3D cartilage assessment is feasible. Like the T2 mapping technique, T2* mapping reflects bulk water content and interactions between water molecules and collagen fibers within cartilage (53). Correspondingly, a characteristic pattern of T2* values with higher numbers in the superficial zone (somewhat related to high-water content and superior water molecule mobility), and lower T2* values toward the cartilage–bone interface (where the uniform perpendicular collagen fiber orientation and high-proteoglycan content endorse water molecule restriction and T2/T2* decay) is noted in normal articular cartilage (66). Nevertheless, distinct differences between these two techniques should be outlined (77). T2 mapping utilizes a spin-echo sequence that comprises a 180° spin re-phasing RF pulse to compensate for local magnetic field inhomogeneities. In brief (51, 53, 78), local magnetic field inhomogeneities cause some spins of individual nuclei to slow down because of lower local field strength, whereas other spins speed up because of higher field strength. This leads to spin dephasing and T2 signal decay. The applied 180° pulse causes the spins to rotate 180°, so that the slower spins are ahead and the fast ones trail behind. Subsequently, the fast spins catch up with the slow spins (re-phasing), eventually regenerating the T2 signal. In contrast, T2* mapping is performed with a GRE technique that lacks the 180° refocusing pulse. Therefore, dephasing effects related to local MR field variations that originate from diverse magnetic susceptibilities among various tissues, chemical shifts and main magnetic field heterogeneities are added to the net T2 decay that explains the characteristically lower T2* values when compared with the T2 measures. These differences have several implications. Because only one RF pulse is applied in GRE-based T2* mapping, the echo can be recorded more rapidly, promoting fast imaging. Furthermore, due to higher echo times (TE) in spin-echo sequences (TE ~10–100 ms), the T2 mapping technique reflects to a large extent the relaxation of bulk water, whereas T2* mapping (with shorter TEs) comprises a wider range of T2 relaxation in cartilage tissue, including signals that decay below 10 ms. T2* mapping is also less susceptible to stimulated echoes and magnetization transfers because it lacks the 180° refocusing pulse. However, enhanced susceptibility effects, such as those related to post-surgical debris or unfavorable anatomic circumstances (for example, closely approximated tissue interfaces), can potentially impair T2* articular cartilage assessment.

### Literature review

**Figure 8:**
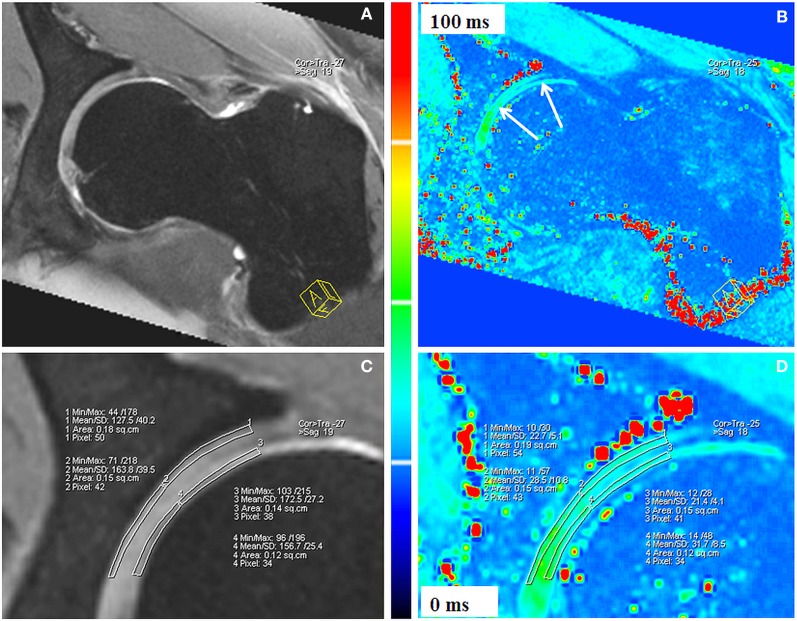
Double-echo steady state (DESS; A,C) and corresponding T2* reformat (B,D) of an asymptomatic volunteer. Sufficient image resolution could be achieved to delineate the cartilage layers of the acetabulum and the femoral head for ROI analysis within peripheral acetabular cartilage, central acetabular cartilage, peripheral femoral cartilage, and central femoral cartilage. The DESS reformats **(A,C)** served as reference for accurate placement of the ROI squares within cartilage. T2* values are illustrated in a color scale **(B,D)**. Figure reprinted with permission (80).

T2* mapping of hip joint cartilage was first reported in 2009 (79). In this pilot study, Bittersohl et al. demonstrated the feasibility of 3D GRE-based T2* mapping at 1.5 T with radial evaluation to assess degenerative changes of hip joint cartilage throughout the hip joint. This study, which enrolled 33 patients with FAI, revealed a significant drop of the T2* values in degenerated cartilage. Limitations of the study included the inability to differentiate clearly between acetabular and femoral head cartilage. The bulk T2* values that were obtained included both acetabular and femoral head cartilage as one entity including the interspersed joint fluid, particularly in areas of severe cartilage damage, which may have caused overestimation of the measured T2* values. This issue was resolved in a follow-up study at 3 T (80) in which a sufficient image resolution could be achieved to delineate the cartilage layers of the acetabulum and the femoral head (Figure 8). In accordance with their previous work, this study group was able to identify a decrease of the T2* values with increasing morphologically apparent cartilage damage (*P* < 0.001) in 29 patients with FAI. Notably, the collected data of 35 healthy, asymptomatic volunteers provided normative T2* values of hip joint cartilage for subsequent studies.

Apprich et al. performed T2* mapping in the acetabular cartilage of 22 patients with clinical signs of FAI (no or mild signs of degeneration in AP radiographs) and 27 age-matched, asymptomatic volunteers at 3 T shortly after the beginning of MRI (early unloading) and after a period of 45 min (late unloading) (81). Although comparison between the T2* values of FAI patients $\left(T2_{\mathrm{global}}^{*}=21.5\pm 3.0\mathrm{ms}\right)$ and ­volunteers $\left(T2_{\mathrm{global}}^{*}=21.8\pm 2.4\mathrm{ms}\right)$ did not reveal any difference after early unloading (*P* = 0.747), significant differences between the T2* values of patients $\left(T2_{\mathrm{global}}^{*}=21.1\pm 2.9\mathrm{ms}\right)$ and those of volunteers $\left(T2_{\mathrm{global}}^{*}=24.6\pm 3.1\mathrm{ms}\right)$ were noted after 45 min of unloading. Notably, the T2* mapping values increased with unloading over time in the control group $\left(T2_{\mathrm{global}}^{*}=21.8\pm 2.4\mathrm{versus}24.6\pm 3.1\mathrm{ms};P=0.001\right)$, whereas a slight decreasing trend was observed for FAI patients $\left(T2_{\mathrm{global}}^{*}=21.5\pm 3.0\mathrm{versus}24.1\pm 2.9\mathrm{ms};P=0.080\right)$.

Siebenrock et al. conducted an experimental ovine FAI model study in which a cam-type FAI was created in eight alpine sheep by performing a closed wedge intertrochanteric varus osteotomy prior to sacrifice 10–14 weeks after surgery and MRI of the hip at 3 T (82). By measuring T2 and T2* values in six locations on the acetabulum (posterior–superior, cranial, anterior–inferior; in each case, centrally and peripherally) and comparing them with histological grades, they found a negative correlation between the histological grading of degenerated cartilage (Mankin grading) and the T2 (*r* = −0.79; *P* < 0.001) and T2* (*r* = −0.90; *P* < 0.001) values. A positive predictive value of 100% and a negative predictive value of 84% were observed for the T2 mapping technique, whereas the T2* technique revealed a positive predictive value of 100% and a negative predictive value of 94%. Topographical T2 and T2* variations were also noted (low values posterior–superior and anterior–inferior at the periphery of the acetabulum).

The most recent report on articular hip joint cartilage assessment by means of T2* mapping in patients suffering from FAI enrolled 28 hips (26 patients) (83). In this retrospective study, the authors correlated T2* maps of acetabular cartilage (superficial, deep, and full-thickness cartilage) with intra-operative arthroscopic cartilage assessment (cartilage degeneration grading according to a modified Beck scale). In this study, lower T2* values were noted for superficial, deep, and full-thickness cartilage in regions with intra-operatively identified cartilage damage (T2* = 20.7 ± 6.0 ms) compared with intra-operatively apparently normal cartilage (T2* = 35.3 ± 7.0 ms, *P* < 0.001). Furthermore, receiver operating characteristic curve analysis (ROC) revealed a threshold T2* value of 28 ms as the threshold for damaged cartilage (91% true-positive and 13% false-positive rate for differentiating normal from abnormal cartilage). Notably, although hip joint arthroscopy was restricted to patients with Tönnis grades 0 and 1, 360 of 532 (68%) regions demonstrated evidence of cartilage damage during arthroscopy. This (again) demonstrates (1) the unreliability of plain radiographs in determining the extent of cartilage damage and (2) the ability of T2* mapping to aid accurate diagnosis of damaged intra-articular cartilage in FAI that could improve our ability to offer a fairly reliable and predictable prognostication of joint status and the appropriateness of intervention in terms of joint preservation or joint replacement.

## Pearls and Pitfalls

Given that the femoral head and acetabular cartilage layers are relatively thin (~1–3 mm each in the weight-bearing zone in a normal hip) (84), spherical in shape and quite closely approximated, quantitative assessment of hip joint cartilage is limited by its relative proneness to chemical shift, susceptibility to artifacts, and volume averaging (fitting of square pixels to a curved structure and, thus, averaging hyaline cartilage with subchondral bone or intra-articular fluid). This is particularly so when the imaging plane is not perpendicular to the curvature of the cartilage. The bulk mapping values of the articular cartilage and the intra-articular space comprise the signal of both articulating cartilage surfaces and the intra-articular joint fluid. This may be reasonably acceptable for visualization purposes. However, in terms of cartilage relaxation time quantification, it leads to erroneous measurements that are pronounced in regions with cartilage abrasion (for example, underestimation of the T1_Gd_ values and overestimation of the T2/T2* values). We, therefore, recommend adjusting the image settings for superior cartilage image quality with high-cartilage contrast and image resolution to achieve optimal cartilage delineation. High-spatial resolution mapping in 2D or 3D radial imaging planes, which allows the orthogonal display of the acetabular cartilage around its circumference, can reduce volume averaging as it provides a true cross-section of the cartilage. Notably, although the generation of 2D radial planes in the hip may be challenging, 3D volumetric acquisitions can be radially reformatted relatively easily. Higher field strengths (≥3 T) in combination with a dedicated and reasonably small surface coil will increase the SNR. The coil should enclose the hip joint as the SNR decays considerably if the distance between the ROI and the coil exceeds the capacity of the device although it is understood that this may pose a challenge in obese patients. A tolerable acquisition time and appropriate patient positioning to avoid motion artifacts must also be considered. Select biochemical MRI parameters currently utilized for *in vivo* hip joint cartilage assessment are summarized in Table 1.

**Table 1:** Selected imaging parameters of previously reported studies of dGEMRIC, T1ρ, T2, and T2* assessment of hip joint cartilage.

	Zilkens et al. (35, 44)	Subburaj et al. (54)	Watanabe et al. (70)	Bittersohl et al. (80)
MRI technique	dGEMRIC	T1ρ mapping	T2 mapping	T2* mapping
Imaging parameters				
Field strength (T)	3	3	3	3
Repetition time, TR (ms)	15	n/s	1500	38
Echo time, TE (ms)	2.24	0, 15, 30, 45	10.3–103	4.62, 9.41, 15.28, 21.15, 27.02, 32.89
Flip angle (°)	5, 26	n/s	n/s	25
Number of excitation	1	n/s	1	1
Field of view (mm)	192	140	150	192
Slice thickness (mm)	0.6	4	4	0.6
In-plane resolution (mm)	0.6 × 0.6	0.5 × 0.5	1 × 1	0.6 × 0.6
Slice gap (mm)	0.12	None	None	0.2
Bandwidth (Hz/pixel)	260	62.5 × 10^3^	315 × 10^3^	260
Acquisition time (min)	14.31	13.40	17.41	13.29

*n/s, not specified*.

Cartilage loading, which may vary locally, has an influence on the extracellular matrix (for example, water outflow because of cartilage compression) (70, 85). This certainly has an impact on the mapping values, and therefore, it is recommended that biochemical MRI should be performed at the end of the MR scan in the (standardized) unloaded state (68, 86). With regard to dGEMRIC, a certain time frame between the contrast agent administration and the T1_Gd_ relaxation time measurement is required to obtain an appropriate cartilage penetration of the gadolinium contrast agent. Regarding dGEMRIC of hip joint cartilage, a time frame of 30–90 min after intravenous application (27) or 15–30 min after intra-articular injection (28) is recommended. The same applies for a reproducible protocol of hip joint motion prior to the T1_Gd_ mapping to enhance appropriately and consistently the gadolinium circulation and uptake within articular cartilage.

Anatomic, inter-subject, and technical variations, such as alterations in acquisition and fitting parameters that can lead to possible misinterpretations with added limited comparability, need to be considered when cartilage-mapping values are read. For example, there are normal regional differences in the composition, ultrastructure, biological activity, and sectoral joint biomechanics of hip joint cartilage (87) that have an influence on the mapping values (for example, higher T1_Gd_ values toward the superior zone reflecting a high-GAG concentration at this weight-bearing region) (25, 39, 42), thereby emphasizing the need for regional analysis of hip joint cartilage. Furthermore, when T2 and T2* mapping is performed in spherically arched cartilage regions, T2/T2* elongation occurs near the so-called “magic angle” of 54.7° relative to the static magnetic field (B_0_) (88). Some observers try to obtain “normalized” regional mapping values by dividing these with some reference value (43). This patient-driven normalization somewhat compensates for deviations caused by technical alterations (e.g., effects of different hardware components and imaging settings, infiltration rate of various dGEMRIC protocols) and variations in the extracellular matrix related to age and individual cartilage configuration. Because many FAI chondrolabral lesions typically originate around the acetabular rim before they progress over time to involve the adjacent cartilage, some researchers suggest that the reference mapping values could be obtained from the central region of the femoral cartilage (34, 36). Notably, despite having advantages, such as short acquisition times, high image resolution and the ability to carry out isotropic 3D cartilage evaluation, GRE-based mapping techniques do lack the 180° refocusing pulse, and therefore, they are more sensitive to local magnetic inhomogeneities (origin of susceptibility artifacts) at the bone–cartilage interface or near artificial particles, such as post-surgical debris and orthopedic implants (53). This effect can substantially compromise the mapping of articular cartilage in postoperative studies. In essence, the mapping values should always be interpreted in conjunction with patient history, clinical examination, and morphological MRI evaluation. In addition, co-existing pathologies, such as hip dysplasia, neoplastic synovitis, bone marrow changes, stress fracture, gluteal enthesopathy, ischiofemoral impingement, advanced (secondary) OA, and several others, may be diagnosed in conjunction with FAI and should be appropriately addressed. FAI may also be bilateral even if only one hip is symptomatic at the time of presentation. Conversely, FAI morphology does not necessarily equate to symptomatic (pathological) FAI and so the exact point of transition remains an enigma.

Finally, despite several studies that have specified the advantages or disadvantages of various cartilage-mapping techniques and their contribution to enhancing cartilage status assessment, biochemically sensitive MRI is still in its infancy. A notable drawback today is the limited applicability of threshold values, as they are dependent on anatomic, inter-subject, and technically related variations and the current lack of clinical correlation. To date, no conclusive imaging data exist for determining an ideal cut-off value for or against surgery in an FAI patient. In the future, it is possible that the ability of these techniques to evaluate cartilage degeneration accurately and reproducibly could improve our ability to offer fairly reliable and predictable prognostication in individual cases for clinical decision-making and treatment.

## Conclusion

Symptomatic FAI occurs from dynamic mechanical conflict between the proximal femur and acetabulum. Since symptomatic FAI is a pre-arthritic condition, early diagnosis and imaging of the relevant patho-anatomy with treatment is important in changing clinical course of early arthritis. Decision-making in symptomatic FAI largely depends on the reliable evaluation of damage to chondrolabral and sectoral articular cartilage, which determines the eventual outcome. Advanced biochemically sensitive MRI techniques, such as dGEMRIC, T2, T2*, and T1ρ mapping, can distinguish subtle early cartilage matrix alterations, thereby acting as tools for early disease detection and monitoring. Despite mapping variations that mirror anatomical differences in various zones and regions of hip joint with these advanced techniques, there are still many unanswered questions including the standardized application of these techniques and cut-off values to provide an algorithmic cartilage damage-based approach to managing FAI. Therefore, further studies that address protocol issues regarding these techniques for the reproducible, objective, and meaningful evaluation of articular hip joint cartilage are necessary. Sufficiently powered, controlled cross-sectional, and longitudinal studies will help to provide cut-off values in order to delineate an appropriate time-point of intervention that could lead to an improved and more predictable outcome. Additionally, improvements in speed, resolution, and applicability will, hopefully, lead to widespread adoption of these techniques. Finally, biochemically sensitive MR imaging could someday help bridge the gap in understanding when does asymptomatic FAI morphology eventually turn into FAI pathology.

## Conflict of Interest Statement

No author and no institution at any time received payment or services from a third party for any aspect of the submitted work. There is no financial relationship with entities that could be perceived to influence, or that give the appearance of potentially influencing, what we wrote in the submitted work. There are no patents and copyrights pending, issued, licensed, and/or receiving royalties relevant to the work. There are no other relationships or activities that readers could perceive to have influenced, or that give the appearance of potentially influencing, what we wrote in the submitted work.
